# Impact of nutritional support that does and does not meet guideline standards on clinical outcome in surgical patients at nutritional risk: a prospective cohort study

**DOI:** 10.1186/s12937-016-0193-6

**Published:** 2016-08-19

**Authors:** Da-Li Sun, Wei-Ming Li, Shu-Min Li, Yun-Yun Cen, Yue-ying Lin, Qing-Wen Xu, Yi-Jun Li, Yan-Bo Sun, Yu-xing Qi, Ting Yang, Qi-Ping Lu, Peng-Yuan Xu

**Affiliations:** 1 Department of General Surgery, Wuhan Clinical School of Southern Medical University/Wuhan General Hospital of Guangzhou Military Command, Wuhan, 430070 China; 2 Department of Gastrointestinal Surgery, Second Affiliated Hospital of Kunming Medical University, Kunming, 650101 China; 3 Research Center for Surgical Clinical Nutrition in Yun-Nan Province, Kunming, 650101 China

**Keywords:** Nutritional risk, Outcome, Guidelines, Abdominal surgery patients, Logistic regression analysis

## Abstract

**Objective:**

To investigate the impact of nutritional support on clinical outcomes in patients at nutritional risk who receive nutritional support that meets guideline standards and those who do not.

**Methods:**

This prospective cohort study enrolled hospitalized patients from the Second Affiliated Hospital of Kunming Medical University from February 2010 to June 2012. The research protocols were approved by the university’s ethics committee, and the patients signed informed consent forms. The clinical data were collected based on nutritional risk screening, administration of enteral and parenteral nutrition, surgical information, complications, and length of hospital stay.

**Results:**

During the study period, 525 patients at nutritional risk were enrolled in the cohorts. Among patients who received nutritional support that met the guideline standards (Cohort 1), the incidence of infectious complications was lower than that in patients who did not meet guideline standards (Cohort 2) (17.1 % vs. 26.9 %, *P* = 0.01). Subgroup analysis showed that individuals who received a combination of parenteral nutrition (PN) and enteral nutrition (EN) for 7 or more days had a significantly lower incidence of infectious complications (*P* = 0.001) than those who received only PN for 7 or more days or those who received nutritional support for less than 7 days or at less than 10 kcal/kg/d. Binary logistic regression analysis showed that, after adjusting for confounding factors, nutritional support that met guideline standards for patients with nutritional risk was a protective factor for complications (OR: 0.870, *P* < 0.002).

**Conclusions:**

In patients at nutritional risk after abdominal surgery, nutritional support that meets recommended nutrient guidelines (especially regimens involving PN + EN ≥ 7 days) might decrease the incidence of infectious complications and is worth recommending; however, well-designed trials are needed to confirm our findings. Nutritional support that does not meet the guideline standards is considered clinically undesirable.

## Background

Nutritional support has been in clinical use for approximately 40 years, and several countries have developed specific nutritional guidelines [1, 2]. However, in practice, many patients still receive nutrients that do not meet guideline standards, including the use of amino acids or fat emulsion only or nutritional support of insufficient amount or duration [3–5].

The reference standards for the amount of energy, nitrogen, and trace elements recommended by nutritional support guidelines are based on the theoretical amounts suitable to ameliorate a human disease state [1, 2]. Nutritional support that meets guideline standards leads to significantly better improvements in clinical outcome than no support in patients who are at nutritional risk or are malnourished [6–8]. However, permissive low-nutrient intake has been shown to result in better clinical outcomes than standard nutritional support in patients after stomach surgery and is considerably less costly [9]. The question of whether nutrition support with nutrient intake between no nutritional support and permissive low-nutrient intake (defined as nutritional support that does not meet guideline standards) can result in clinical outcomes comparable to those of patients who receive full nutritional support has not been addressed.

Current nutritional support guidelines do not explicitly state how long perioperative nutritional support should be administered to patients at nutritional risk. Although certain guidelines and related studies have suggested that patients with severe preoperative malnutrition should receive total parental nutrition (TPN) for at least seven days to improve clinical outcomes [1, 8], this is clinically unrealistic for most patients at nutritional risk. This approach can delay surgery, prolong hospital stays, and increase hospital costs, placing it at odds with the concept of ethics and health care reform. Consequently, postoperative nutritional support is currently used in clinical practice, and nutritional support lasting at least 7 days is thought to be effective. Nutritional support for less than 7 days is considered inadequate to meet guideline standards.

There is a need for research comparing the impact of nutritional support on clinical outcomes in at-risk patients who receive guideline-recommended nutritional support and those who do not. Although a randomized controlled study can provide high-level evidence with which to evaluate clinical interventions, there are serious ethical issues in designing and implementing a randomized controlled study that compares clinical outcomes between patients who receive recommended levels of nutritional support guideline standardsand those who do not. A rigorous cohort study was therefore considered the most feasible approach for the current study. The reality is that some Chinese clinicians in certain parts of China have yet to fully implement nutritional support in accordance with standardized nutrition guidelines, and some departments cannot adequately prepare the standard nutrient solutions due to poor hospital conditions. This situation creates two de facto treatment groups of patients at nutritional risk who are receiving nutrients: those receiving full nutritional support as stipulated in guidelines and those receiving partial support. Partial support may involve fewer calories, less nitrogen, or a shorter duration of nutritional support (<7 days) than recommended. These two groups make it possible to conduct a cohort study at this stage among patients receiving nutritional support that does not meet guideline-recommend standards.

This prospective cohort study was designed to compare the impact of nutritional support on clinical outcome in patients at nutritional risk who had undergone major abdominal surgery and received guideline-recommended nutritional support (involving two nutritional support programs, parenteral nutrition [PN] and a combination of PN and enteral nutrition [EN]) and those who did not (in two categories, those who received <10 kcal/kg/d and those whose nutritional support lasted <7 days).

## Methods

This prospective cohort study was registered at www.chictr.org, identifier ChiCTR-OOC-16008782, and approved by the ethics committee at the Second Affiliated Hospital of Kunming Medical University (approval no. 2010-08). The study was performed in accordance with the Declaration of Helsinki [10]. All patients enrolled in the study provided informed consent.

The study enrolled patients from 5 general surgery departments with a total of 200 beds at the Second Affiliated Hospital of Kunming Medical University. Continuous sampling was performed to collect research data from February 2010 to June 2012.

The study subject inclusion criteria were as follows: (1) undergoing major abdominal surgery confirmed by clinicians, including partial gastrectomy, full stomach resection, colorectal resection, pancreaticojejunostomy, biliary-enteric anastomosis, hepatectomy, biliary exploratory surgery, duodenal resection, and small-bowel resection and being (2) ≥18 years of age, <90 years of age; (3) willing to participate in this study; (4) capable of communicating with researchers; (5) screened for nutritional risk according to Nutritional Risk Screening-2002 (NRS-2002) with a resulting nutritional risk ≥3 points; and (6) hospitalized for more than 7 days.

Study exclusion criteria were as follows: (1) being pregnant or lactating; (2) having impaired cognitive function; (3) not undergoing a face-to-face examination within 48 h of admission; (4) having severe illness or being transferred from the intensive care unit or other medical institutions; (5) undergoing emergency surgery; and (6) receiving more calories or nitrogen than recommended by guidelines or receiving nutritional support for only one day.

The patients were evaluated in person for the first time within 48 h of admission to obtain their informed consent, assess nutritional risk, and collect baseline information.

The inpatient nutritional risk screening method recommended by the European Society of Parenteral and Enteral Nutrition (ESPEN) and the Chinese Society for Parenteral and Enteral Nutrition (CSPEN), NRS-2002, was used within 48 h of admission to screen the nutritional risk of patients who met the inclusion criteria, and patients whose total score was ≥3 were considered at nutritional risk and included in the follow-up programme.

All patients who completed the first assessment and met the selection criteria received follow-up 4 times a week until discharge to ensure the prospective nature of the study. Each patient’s medical records, nursing records, and face-to-face interviews were used to gather information about their complications and nutritional support and to record the discharge date and discharge outcomes. In all cases, the assessment and collection of information were completed by specialized researchers with the help of doctors and nurses in the inpatient department.

### Justification and definition of nutritional support

In this study, the nutritional support programme was selected by the patients’ attending physicians rather than being randomly determined or affected by the researchers. The physicians’ selection of nutritional support programme was determined primarily by the following factors: (1) the physician’s clinical nutrition knowledge; (2) attitudes toward and awareness of nutritional support of the patients and their families; (3) Medicare coverage; and (4) nutritional support routinely practiced by the department. Because the attending physicians were blind to the nutritional risk screening results, the results did not affect their selction of nutritional support programme.

During the study period, the physicians at the Second Affiliated Hospital of Kunming Medical University had not yet started following the clinical practice guidelines for nutritional support recommended by the CSPEN. Although the hospital had nutritional support and consultation groups and nutrition-preparation rooms to provide standard nutritional technology and support, some clinicians and departments did not use the prepared nutrient solution and followed departmental or personally preferred methods of nutritional support, such as administering insufficient quantity of amino acids, fat emulsion alone or support for a limited duration. For the subjects included in this study, different levels of nutrients (at a minimum, glucose and amino acids) were administered instead of a glucose saline solution (i.e., no nutritional support).

The ESPEN and CSPEN standards were used to define the concept of nutritional support.

PN: The ESPEN and Chinese Medical Association Clinical Practice Guidelines served as the reference. PN was defined as administration of sugar/fat and amino acids at a dose of 15–30 kcal/kg/d (non-protein) and ≥0.8 g/kg/d of amino acids, with continuous application for ≥7 days at intervals of no more than 48 h [1, 2, 9]. The PN formulation used in this study was in a three-chamber bag (Cavan Kabiven, Huarui Pharmaceuticals, Wuxi, China) and prepared in a sterile room in the hospital (TPN preparation room). In this study, PN was administered through the surrounding central vein/central vein (internal jugular vein/subclavian vein). Continuous infusion of PN lasted at least 16 h a day.

EN was also administered in accordance with the ESPEN and the Chinese Medical Association Clinical Practice Guidelines. The treatment, which was administered via a parenteral route (oral or tube feeding), consisted of industrialized enteral nutrition, such as whole protein and peptides, including nutritional products from SSPC and Nutricia. The calories were maintained at 15–30 kcal/kg/d (≥1 g/kg/d of protein), with continuous adminstration for ≥7 days at intervals of no more than 48 h of receiving EN [2, 9, 11]. The continuous tube feeding time was at least 16 h per day, with 100 ml per hour of oral EN.

The combination of PN and EN consisted of the above-mentioned PN and EN methods, and the total number of calories could reach 15–30 kcal/kg/d (≥0.8 g/kg/d of amino acids), with continuous administration for ≥7 days, at intervals of no more than 48 h. If the patients also continued normal dietary intake, the parenteral or enteral nutrition support would provide more than 80 % of the patient’s total energy to meet the requirements described above and to be included in the nutritional support cohort.

Nutrient supplementation programmes did not meet the recommended guidelines if (1) the calories or proteins in the PN, EN, or PN + EN met the standards but the treatment lasted ≤6 days or (2) the calories or nitrogen were lower than the PN or EN mentioned above but lasted ≥2 days.

### Complications

The most common complications after abdominal surgery in the patients were pneumonia, septicaemia, intra-abdominal abscess, wound infection, urinary tract infection, central catheter infection, anastomotic blood, anastomotic leakage, wound dehiscence, intestinal obstruction, organ dysfunction, pulmonary embolism, bleeding due to duodenal ulcer, abdominal bleeding, severe electrolyte imbalance, deep venous thrombosis and pleural effusion/pneumothorax. The primary endpoint was the incidence of infectious complications. Infectious complications were defined as the appearance of pathogens in the sterile tissue within the body confirmed by pathogen culture results or by evidence of infection corresponding to clinical signs and symptoms, radiological results, or haematology [12]. Infection of the incisional wound was defined as purulent exudate in the wound with a positive bacterial culture. Clinical signs of pneumonia and radiographic evidence and/or a positive bacterial culture of tracheal aspirate, blood or brush were considered to indicate pneumonia. An intra-abdominal abscess was defined as an abscess diagnosed by radiographic examination that required operative or percutaneous drainage. Anastomotic leakage was defined as clinical suspicion of leakage of anastomosis, confirmed by radiographic examination, or a visually dehiscent anastomosis during reoperation. A catheter-related infection was defined as a positive culture of a catheter combined with a positive bacterial culture.

### Postoperative hospital stay

Because nutritional support was administered after surgery in all the included patients, the analysis of postoperative hospital stay could accurately indicate the impact of nutritional support on the length of stay. Postoperative hospital stay was defined as the duration between the date of operation and the date when discharge criteria were met. Discharge criteria included the ability to manage personal care and toilet activities, absence of fever, and no intravenous access [13].

### Sample-size determination

Before the study was initiated, retrospective data from 100 at-risk patients who had undergone major abdominal surgery showed that the infection complication rate among patients who received calories or nutritional support that did not meet guideline standards was approximately 30 %. Nutritional support that did meet guideline standards was considered an efficient method of reducing the incidence of infectious complications. During the study period, an estimated 500 patients at nutritional risk were expected to meet the inclusion criteria. If 2/3 of the patients were given nutritional support that met guideline standards, then, using a 2-sided alpha of 0.05, 500 patients would provide 80 % power to detect a 40 % reduction in infectious complications in the cohort given nutritional support that met guideline standards (15.5 %) relative to the cohort given nutritional support that did not (26 %).

### Statistical analysis

In this study, EpiData software 3.0 was used to establish the database. Excel 2010 was used to organize the data. The *χ* ^2^ test and Fisher’s exact test were used to compare the categorical variables. Normally distributed data were expressed as the mean ± SD, and non-normally distributed variables were expressed as medians and interquartile ranges (IQRs). For continuous variables, the distribution of the data was analysed for normality. Student’s unpaired *t*-test was used for normally distributed numerical variables, and Wilcoxon test was used for non-normally distributed numerical variables. Binary logistic regression analysis was used to analyse the risk factors (gender, age, BMI, weight loss, reduced food intake, nutrition support, energy and nitrogen) for infectious complications. SPSS18.0 (SPSS Inc, Chicago, IL, US) software was used for the statistical analysis. *P* < 0.05 indicated statistical significance, and *P* < 0.01 indicated statistical significance.

## Results

### General information regarding the study subjects

**Fig. 1:**
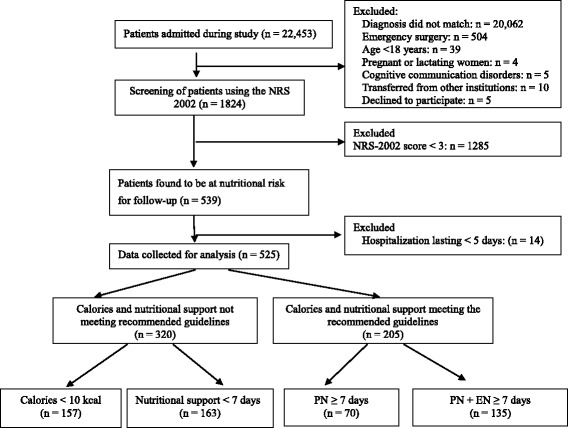
Flowchart of subject inclusion process

During the study period, there were a total of 22,453 patients in the five wards, and the inclusion process was as described in the flowchart (Fig. 1). Of the 1824 patients who were treated with NRS 2002, 1285 with NRS-2002 scores <3 were excluded; 539 patients were found to be at nutritional risk, and 14 of these patients were excluded because they were hospitalized for <5 days. Ultimately, 525 patients at nutritional risk were analysed in the study. Although nutritional support should have been administered to patients at nutritional risk (NRS-2002 score ≥3), only 39.05 % (205/525) of such patients received nutritional support that met the recommended guidelines. The other 60.95 % received caloric and nutritional support that did not meet recommended guidelines (320/525).

The demographic characteristics and baseline information for the subjects are shown in Table 1. Significant differences were found between cohorts in gender, age, body weight loss >5 %, average calories, average amount of N and diagnosis (*P* < 0.05). There were no differences in nutritional risk, BMI, food intake < 75 %, or postoperative hospital stay between cohorts (*P* ≥ 0.05).

**Table 1:** Baseline characteristics of patients in cohorts receiving and not receiving nutritional support meeting guideline standard

	Calories or nutritional support not meeting guideline standards (*n* = 320)	Calories and nutritional support meeting guideline standards (*n* = 205)	*P* value
Male (percentage)	188 (58.7 %)	100 (48.8 %)	0.025
Age (mean ± SD)	66.43 ± 11.73	62.86 ± 13.99	0.006
Nutritional risk (mean ± SD)	4.09 ± 0.90	4.08 ± 0.93	0.134
BMI (mean ± SD)	21.09 ± 2.51	20.80 ± 2.56	0.687
Body weight loss >5 %	126 (39.4 %)	117 (57.1 %)	<0.001
Food intake <75 %	240 (75.0 %)	142 (69.3 %)	0.150
Average calories (kcal/person/day)	15.35 ± 2.44	22.67 ± 1.48	< 0.001
Average N amount (g/person/day)	0.66 ± 0.51	1.15 ± 0.09	< 0.001
Duration of nutritional support (days)	——	10.5 ± 2.6	——
Postoperative hospital stay (days)	15 (13,19)	15 (12, 20)	0.609
Diagnosis^a^ (n)			< 0.001
A1	11 (3.4 %)	6 (2.9 %)	
A2	123 (38.4 %)	117 (57.1 %)	
B1	129 (40.3 %)	60 (29.3 %)	
B2	57 (17.8 %)	22 (10.7 %)	

^a^ A1 = benign gastrointestinal diseases: gastric ulcer, duodenal ulcer, gastric stromal tumours, jejunum stromal tumours, congenital megacolon, lengthy colon, rectal polyps, colonic diverticulitis; A2 = gastrointestinal cancer: gastric cancer, colon cancer, colorectal cancer, duodenal cancer, small intestine cancer, non-Hodgkin’s lymphoma; B1 = benign disease of the liver, bile ducts and pancreas: bile duct stones, bile duct stricture, Mirizzi syndrome, bile duct cyst, liver haemangioma, pancreas cystadenoma, papillary tumour of the pancreas, pancreatic pseudocyst; B2 = malignant cancer of the liver, bile ducts and pancreas: cholangiocarcinoma, gallbladder cancer, liver cancer, pancreatic cancer

### Complications

Differences in complications in the 2 cohorts are shown in Table 2. Of the 525 patients at nutritional risk, 126 experienced complications: 121 of these were infectious complications and 17 were non-infectious (including 12 cases of co-infection complications). Caloric or nutritional support that met recommended guidelines was associated with a significantly lower incidence of total complications than caloric and nutritional support that did not meet the recommended guidelines (17.6 % vs. 28.1 %, *P* = 0.006). The incidence of infectious complications was lower (17.1 %) in the cohort given recommended levels of caloric or nutritional support than in the cohort that did not (26.9 %) (*P* = 0.009). There were no differences in non-infectious complications or mortality between the cohorts (*P* ≥ 0.05). Eight patients died: 3 with multiple-organ dysfunction, 1 with anastomotic leakage, 3 with lung infections and uremic syndrome, and 1 with septic shock.

**Table 2:** Differences in complications between cohorts receiving and not receiving nutritional support meeting guideline standard

	Calories or nutritional support not meeting guideline standards (*n* = 320)	Calories or nutritional support meeting guideline standards (*n* = 205)	*P* value
(1) Infectious complications	86 (26.9 %)	35 (17.1 %)	0.009
Pneumonia	52 (16.3 %)	18 (8.8 %)	0.014
Urinary tract infection	4 (1.3 %)	0 (0.0 %)	0.160
Sepsis	25 (7.8 %)	10 (4.9 %)	0.187
Septic shock	0 (0.0 %)	2 (1.0 %)	0.152
Catheter-related infection	1 (0.3 %)	0 (0.0 %)	1.000
Intra-abdominal abscess	6 (1.9 %)	10 (4.9 %)	0.051
Infection of incisional wound	11 (3.4 %)	1 (0.5 %)	0.034
Two or more infections	12 (3.8 %)	7 (3.4 %)	1.0
(2) Non-infectious complications	8 (2.5 %)	9 (4.4 %)	0.312
Organ dysfunction	2 (0.4 %)	1 (0.5 %)	1.000
Multiple organ failure	5 (1.6 %)	1 (0.5 %)	0.412
Pulmonary embolism	1 (0.3 %)	0 (0.0 %)	1.000
Bleeding due to duodenal ulcer	3 (0.9%)	0 (0.0 %)	0.285
Postoperative abdominal bleeding	1 (0.3 %)	1 (0.5 %)	1.000
Severe electrolyte imbalance	1 (0.3 %)	1 (0.5 %)	1.000
Anastomotic bleeding	1 (0.3 %)	0 (0.0 %)	1.000
Anastomotic leak	0 (0.0 %)	2 (1.0 %)	0.152
Pleural effusion/pneumothorax	0 (0.0 %)	1 (0.5 %)	0.390
Wound dehiscence/healing delay	0 (0.0 %)	1 (0.5 %)	0.390
Gastrointestinal obstruction/perforation	0 (0.0 %)	1 (0.5 %)	0.390
Deep vein thrombosis	1 (0.3 %)	1 (0.5 %)	1.000
Two or more non-infectious complications	5 (1.6 %)	1 (0.5 %)	0.412
Co-infectious complications	4 (1.3 %)	8 (3.9 %)	0.069
(3) Total complications	90 (28.1 %)	36 (17.6 %)	0.006
(4) Deaths	6 (1.9 %)	2 (1.0 %)	0.492

Chi-square tests or Fisher’s exact test were used

The differences in complications between the 4 subgroups are shown in Table 3. There were significant differences between subgroups with respect to infectious complications, non-infectious complications, total complications and mortality rate (*P* < 0.05). The Bonferroni method was used to account for multiple comparisons among subgroups. The incidence of infectious complications and total complications was lower (12.6 %) in the subgroup receiving PN and EN combined nutritional support for ≥7 days than in the other subgroups (*P* < 0.05). The incidence of non-infectious complications was higher (8.6 %) in the subgroup receiving PN for ≥7 days than in the other subgroups (*P* < 0.05).

**Table 3:** Incidence of complications among subgroups

	Calories or nutritional support not meeting guideline standards (*n* = 320)		Calories or nutritional support meeting guideline standards (*n* = 205)		*P* value
	Nutritional support < 7 days (*n* = 163)	Calories < 10 kcal (*n* = 157)	PN + EN ≥ 7 days (*n* = 135)	PN ≥7 days (*n* = 70)	
Infectious complications	43 (26.3 %) ^a^	43 (27.4 %) ^a^	17 (12.6 %) ^b^	18 (25.7 %) ^a^	0.010
Non-infectious complications	3 (1.8 %) ^a^	5 (3.2 %) ^a^	3 (2.2 %) ^a^	6 (8.6 %) ^b^	0.050
Total complications	46 (28.2 %) ^a^	44 (28.0 %) ^a^	17 (12.6 %) ^b^	19 (27.1 %) ^a^	0.005
Death	2 (1.2 %) ^a^	4 (2.5 %) ^a^	0 (0.0 %) ^a^	2 (2.8 %) a	0.025

Chi-square tests or Fisher’s exact test were used

^a^denotes a subset of cohort categories whose column proportions did not differ significantly from each other, *P* ≥ 0.05

^b^denotes a subset of cohort categories whose column proportions differed significantly from each other, *P* < 0.05

Binary logistic regression analysis was performed to identify the risk factors for infectious complications (Table 4). Infectious complications served as the dependent variable in the analysis, and gender, age, BMI, weight loss, reduced food intake, type of nutritional support, disease, and type of surgery were the independent variables. Binary logistic regression analysis conducted to identify the factors that influenced infectious complications showed that age, weight loss, and reduced food intake were risk factors for infectious complications and that nutritional support that met recommended guidelines was a protective factor.

**Table 4:** Risk factors for infectious complications

Variable	Parameter	SEM	Wald value	*P* value	OR 95 % CI
Gender	0.224	0.249	0.809	0.368	1.251 (0.768, 2.037)
Age	0.029	0.012	5.963	0.015	1.030 (1.006, 1.055)
BMI	−0.027	0.05	0.284	0.594	0.974 (0.883, 1.074)
Body weight loss >5 %	0.093	0.045	4.306	0.038	1.097 (1.005, 1.198)
Food intake <75 %	0.449	0.19	5.594	0.018	1.567 (1.080, 2.273)
Nutritional support that met recommended guidelines	−0.139	0.045	9.394	0.002	0.870 (0.796, 0.951)
Energy	0.106	0.054	3.807	0.051	1.111 (0.998, 1.236)
Nitrogen	−1.446	0.753	3.685	0.055	0.235 (0.054, 1.031)

Binary logistic regression analysis was used

## Discussion

China is currently undergoing health care reform and improvements to the health care system; standardizing medical management and behaviour in medical institutions and promoting guidelines are two important objectives. Standard nutritional support for hospitalized patients at nutritional risk has been recommended by the Clinical Practice Guidelines [2], but our study showed that the actual situation was not congruent with these recommendations. This prospective cohort study showed that approximately 60.95 % of the patients at nutritional risk who underwent major abdominal surgery did not receive nutritional support consistent with recommended guidelines. Of all the patients in this study, 29.90 % were given only amino acids, at <10 kcal/kg/d, and 31.05 % received nutritional support for less than 7 days. The main reasons for these inadequate procedures were lack of screening for medical nutrition risk and lack of knowledge concerning nutritional support [14, 15]. The need for nutritional support was usually determined by a physician, based on insufficient food intake, hypoproteinemia, or general critical condition [14].

More importantly, we found that nutritional support that did not meet the standards in the guidelines (i.e., nutritional support for < 7 days and calories <10 kcal) was associated with a higher incidence of infectious complications and total complications than guideline-compliant nutritional support. However, it is prudent to qualify this result because possible confounding factors, such as gender, age, body weight loss, average calories, average amount of N and diagnosis, may have introduced bias. To adjust for the confounding factors as much as possible and more precisely evaluate the impact of nutritional support on the incidence of infectious diseases in patients at nutritional risk, binary logistic regression analysis was performed to account for these variables. The analysis showed that nutritional support that met the guideline standards was a protective factor for patients at nutritional risk. However, the OR value was 0.870, and the strength of the correlation was moderate. After adjusting for the confounding factors, higher age, weight loss, and reduced food intake were also found to increase the incidence of infectious complications (Table 4). These results confirmed the effects of nutritional support on clinical outcome and also demonstrated that nutritional deterioration was related to age and poorer clinical outcomes. This is consistent with the findings reported by Kondrup et al. [16].

In previous prospective cohort studies of nutritional support in patients at risk, nutritional support consisted mainly of PN and little to no PN combined with EN; therefore, the investigators were unable to conduct a complete statistical analysis [6, 8, 15]. In the present study, 65.85 % of patients with nutritional support lasting ≥7 days received combined PN and EN nutritional support, which was suitable for data analysis. Previous studies included EN-only groups, mostly for nutritional support administered to patients with internal illness or nutritional support before abdominal surgery. PN was frequently used for postoperative nutritional support after abdominal surgery [6, 8, 15]. In this study, no EN-only groups were identified for the following reasons: (1) physicians lacked adequate knowledge regarding nutrition support and awareness of EN application methods; (2) surgical safety concerns of the clinician, such as administration of intestinal nutritional support after gastrointestinal surgery, increasing anastomotic tension and thus being unfavourable for anastomotic healing; and (3) the need for catheters and careful monitoring of the implementation process, such as adjusting the concentration, speed, and temperature, which is more complicated than the implementation of PN.

In this study, the incidence ratio of infectious complications between the groups given nutritional support that met guideline standards and those that did not was 1:1.6 (17.1 % vs. 26.9 %, *P* = 0.01), which was higher than the incidence ratio in previous studies (approximately 1:2) [6, 14]. The sub-cohort analysis (Table 3) showed a similar rate of infectious disease in patients given PN nutritional support for ≥ 7 days and those whose treatments did not meet the standards recommended in guidelines (i.e., nutritional support <7 days or calories <10 kcal). This was consistent with the conclusion that TPN for ≥7 days does not significantly reduce the rate of infectious complications compared with no nutritional support at all [17]. In the combined EN and PN group, along with other sub-groups of the cohorts (PN ≥ 7 days, nutritional support for <7 days and calories <10 kcal), the incidence of infectious complications was significantly lower, which was considered a benefit conferred by EN. However, these results need to be confirmed and supported by additional trials with sufficient sample sizes.

There are several limitations of this study. First, there were no previous studies to draw on for the calculation of sample size. Because infectious complications were extracted retrospectively from patient records, the results may not have been fully accurate. Second, the categorization of some variables in the logistic regression analysis was too general. Some valuable information may have been missed. Third, logistic regression analysis is notoriously unstable; therefore, the associations we found need to be confirmed in future studies. Although our study had these limitations, our findings might provide a useful reference for Chinese clinicians and policy makers to improve nutritional support practices.

## Conclusions

This study demonstrated that nutritional support that did not meet the standards in recommended guidelines was associated with a higher incidence of infectious complications in patients at risk after abdominal surgery and is thus clinically undesirable. Nutritional support meeting the recommended nutrient guidelines was a protective factor for infectious complications, but the strength of the correlation was moderate. A support regimen involving PN + EN for ≥7 days generated significantly better outcomes and is therefore highly recommended. These positive findings in our study need to be confirmed and supported by additional well-designed trials.

## Abbreviations

CSPEN, Chinese society for parenteral and enteral nutrition; EN, enteral nutrition; ESPEN, European society for parenteral and enteral nutrition; IQR, interquartile range; NRS-2002, nutritional risk screening-2002; PN, parental autrition; TPN, total parental nutrition
